# Evaluating genetic and genomic tests for heritable conditions in Australia: lessons learnt from health technology assessments

**DOI:** 10.1007/s12687-021-00551-2

**Published:** 2021-09-27

**Authors:** Sarah Norris, Andrea Belcher, Kirsten Howard, Robyn L. Ward

**Affiliations:** 1 Menzies Centre for Health Policy and Economics and School of Public Health, Faculty of Medicine and Health, University of Sydney, Sydney, NSW Australia; 2 Australian Genomics, Melbourne, VIC 3052 Australia; 3 Faculty of Medicine, The University of Queensland, Brisbane, QLD 4072 Australia; 4 University of Queensland, Brisbane, QLD Australia; 5 Faculty of Medicine and Health, University of Sydney, Sydney, NSW Australia

**Keywords:** Genomics, Heritable, Health technology, Value, Evaluation, Cost

## Abstract

The Medical Services Advisory Committee (MSAC) is an independent non-statutory committee established by the Australian government to provide recommendations on public reimbursement of technologies and services, other than pharmaceuticals. MSAC has established approaches for undertaking health technology assessment (HTA) of investigative services and codependent technologies. In 2016, MSAC published its clinical utility card (CUC) Proforma, an additional tool to guide assessments of genetic testing for heritable conditions. We undertook a review and narrative synthesis of information extracted from all MSAC assessments of genetic testing for heritable conditions completed since 2016, regardless of the HTA approach taken. Ten assessments met our inclusion criteria, covering a range of testing methods (from gene panels to whole-exome sequencing) and purposes (including molecular diagnosis, genetic risk assessment, identification of congenital anomaly syndromes, and carrier screening). This analysis identified a range of methodological and policy challenges such as how to incorporate patient and societal preferences for the health and non-health outcomes of genomic testing, how best to capture the concept of co-production of utility, and how to engage clinicians as referrers for genomics tests whilst at the same time ensuring equity of access to a geographically dispersed population. A further challenge related to how qualitative assessments of patient and community needs influenced the evidence thresholds against which decisions were made. These concepts should be considered for incorporation within the value assessment frameworks used by HTA agencies around the world.

## Introduction

Health technology assessment (HTA) is a multidisciplinary process that uses explicit methods to determine the value of a health technology at different points in its lifecycle, for the purpose of informing decision-making that promotes an equitable, efficient, and high-quality health system (O'Rourke et al. 2020). A health technology is any intervention developed to prevent, diagnose or treat medical conditions, promote health, provide rehabilitation, or organise healthcare delivery. A health technology can be a test, device, medicine, vaccine, procedure, service, program, or system.

HTA is a formal, systematic, and transparent process that uses state-of-the-art methods to consider the best available evidence (O'Rourke et al. 2020). An HTA typically considers the following: the health problem and current use of the technology; a description of the technology and its technical characteristics; safety; clinical effectiveness; an economic evaluation; an ethical analysis; relevant organisational aspects; patient and social aspects; and legal aspects (EUnetHTA 2016).

HTA is undertaken in many countries, by a range of government, not-for-profit, and commercial organisations, and has been used for more than four decades to inform clinical guidance, policy making, and funding (coverage) decisions (Banta and Jonsson 2009). Whilst the general approach to HTA is similar across countries, key differences do arise because of the perspective of the organisation undertaking the HTA, the perspective of the payer, and the features of the local health system (including its public and private health sector funding models).

## Health technology assessment in Australia

In Australia, there are various funding schemes that provide public subsidy for pharmaceuticals, vaccines, medical services, devices, and tests. Reimbursement via public subsidy is assessed by one of three committees, each of which adopts an HTA approach. Assessment occurs after the national regulatory authority has approved the technology for marketing. One of these HTA committees, the Medical Services Advisory Committee (MSAC), is responsible for assessing genetic and genomic tests (see below). Requests for public subsidy must be initiated by a ‘Sponsor’, which can be any organisation or individual, but is typically a product manufacturer, service provider, clinical group, or a government entity. Details of these HTA processes are publicly available (Australian Government Department of Health 2020).

MSAC provides advice to the federal Minister for Health regarding the comparative safety, effectiveness, and cost-effectiveness of a technology. If public subsidy for the technology is supported, one or more items will be established on the Medicare Benefits Schedule (MBS) which describe the service that is subsidised and an MBS fee that reflects the costs and resources required to deliver the service. For a medical test on the MBS, the fee would reflect the consumables and the time of the relevant health professional(s) to collect, prepare, analyse, and report on the biological specimen.

## Health technology assessment of genetic tests in Australia

MSAC has well-established processes and methods for undertaking HTA of medical tests, including genetic tests. The standard MSAC approach for evaluating tests is detailed in technical guidelines for the assessment of investigative services (Medical Services Advisory Committee 2017), which also includes specific guidance for assessing companion diagnostics (referred to in Australia as ‘codependent technologies’). Many assessments of genetic tests for somatic pathogenic single gene variants have been undertaken by MSAC, typically for the purpose of establishing eligibility for subsidised pharmaceuticals, for example, *ALK* rearrangement testing in patients with non-small cell lung cancer to determine eligibility for crizotinib (Medical Services Advisory Committee 2013), or *RAS* mutation testing for eligibility for panitumumab in metastatic colorectal cancer (Medical Services Advisory Committee 2014).

However, early applications to MSAC for genetic tests for heritable conditions highlighted deficiencies in the guidelines for investigative services and codependent technologies. For example, MSAC deferred a funding decision for cystic fibrosis transmembrane regulator testing because issues specific to genetic/genomic testing were not addressed (Medical Services Advisory Committee 2015b). These included eligibility for testing, genetic counselling requirements, the impact of undertaking testing in biological relatives (cascade testing), the role of routine antenatal screening for reproductive planning, and the likely impact of next-generation sequencing (NGS). Similarly, an application for pre-implantation genetic diagnosis (PGD; Medical Services Advisory Committee 2015a) was deferred by MSAC with the committee noting the absence of data on psychological distress from prenatal testing, consumer preference for prenatal testing versus PGD, and the risks associated with termination of pregnancy. MSAC also noted that non-medical considerations, such as psychological, ethical, and social issues, and the management of genetic risk were not adequately addressed.

## Development of a new assessment framework for tests for heritable conditions

MSAC established a Predisposition Genetic Testing Working Group (the Working Group) to develop and pilot an HTA approach for assessing genetic and genomic testing for heritable conditions. The Working Group was convened from 2015 to 2016 and included individuals with a range of experience in clinical or molecular genetics, health economics, MSAC processes and methods, and representatives of the Royal College of Pathologists of Australasia (RCPA). A scoping review of international approaches to the assessment of genetic tests identified the EuroGentest clinical utility gene card (Schmidtke and Cassiman 2010), and the ACCE evaluation process for genetic testing: analytical validity, clinical validity, clinical utility, and ethical, legal, and social implications (Burke et al 2002). The Working Group combined the domains from these two assessment tools with the HTA domain of costs and economic evaluation to develop the clinical utility card (CUC) Proforma (Medical Services Advisory Committee, 2016a). The CUC Proforma was piloted with Application 1411—testing for *BRCA1/2* germline pathogenic variants in people with breast or ovarian cancer—and subsequently revised following re-assessment of this application (i.e. Application 1411.1).

Importantly, the CUC Proforma was constructed from a clinical perspective of disease management, to allow determination of the populations to be tested (affected individuals and their family members) and characterisation of the consequences of testing (potentially over long timeframes). As the name suggests, it places an emphasis on clinical utility, which was defined for MSAC purposes as testing which yields an actionable result which in turn leads to changes in health outcomes in affected individuals and/or their biological relatives. The CUC Proforma introduced three key concepts for HTA undertaken by MSAC: (i) the importance of selecting individuals for testing based on the likelihood that they harbour heritable pathogenic variants (referred to as the pre-test probability of pathologic heritable mutation(s)), (ii) ‘star performer’ genes, and (iii) the co-production of health-related utility that occurs when family members experience positive health outcomes.

MSAC’s preference for an actionable genetic test result was reflected in the CUC Proforma requirement for a pre-test clinical assessment of at least a 10% likelihood of pathogenic variants in the ‘star performer’ gene(s) in the affected individual. This pre-test probability threshold was introduced to avoid testing in populations with a high probability of an uninterpretable or unactionable result (i.e. genetic testing with low clinical utility).

‘Star performer’ genes were defined as high penetrance, actionable gene(s) within a disease area that are likely to have the clearest evidence of clinical utility, and which will consequently have the strongest cost-effectiveness argument. A request for public funding could include additional relevant genes (i.e. genes with sufficient penetrance included in well-regarded clinical practice guidelines), but a lower threshold of evidence would be applied to these. Co-production of health-related utility recognises that any value of cascade testing in family members can only accrue if probands have been tested. This is important for the economic evaluation of cascade testing, as it means the marginal cost-effectiveness of testing family members must be derived from a comparison of the cost-effectiveness of testing affected individuals *and* testing family members of probands *versus* the cost-effectiveness of only testing affected individuals.

## Selecting the approach for an MSAC application

Before applying to MSAC, sponsors have the option of participating in one or more pre-submission meetings with the Department of Health, to discuss how they will frame their application. The selection of a specific assessment approach for an application is not rules based: sponsors are free to choose which approach they will apply, and the investigative services guidelines and codependent technology guidelines can be used for any type of test. For genetic or genomic testing, any one of the three assessment approaches may be appropriate: the CUC Proforma approach when testing is likely to be required in biological relatives as well as index cases; the codependent technologies approach when the results of testing can rule in or rule out the use of a particular pharmaco- or immunotherapy; and the investigative services approach for all other purposes.

Since 2015, each of the three approaches (investigative services, codependent technologies, and the CUC Proforma) has been used to frame assessments of genetic or genomic testing for heritable conditions. The aim of this paper was to review MSAC’s experience assessing tests for this purpose, regardless of the HTA approach used, and share current thinking regarding the need for further evolution of MSAC’s approach to the assessment of genetic and genomic testing for heritable conditions, and for genomic testing more broadly.

## Methods

### Study selection and inclusion criteria

All applications listed on the MSAC website were searched by two authors (AB and SN) to identify potentially relevant applications. Applications were included for review if they met the following criteria: were for a genetic or genomic test for a heritable condition; were considered by MSAC at or after their March 2016 meeting (when the CUC Proforma was available); and there was a public summary document (PSD) available on the MSAC website at the time of the initial (January 2021) or updated search (April 2021). A PSD describes the rationale and funding advice from MSAC to the Minister for Health, together with a summary of the full HTA report. Sometimes information considered by the sponsor to be commercial-in-confidence will be redacted in the PSD.

### Data collection and analysis

For each included application information from the relevant PSD(s) was initially extracted into a data extraction form by AB followed by checking and completion of the data extraction by SN.

The following data were extracted: the defining characteristics of the initial population(s) to be tested; the purpose of testing (as per Korf and Irons 2012); the ‘scale of testing’ (which describes the comprehensiveness of sequencing, from more targeted sequencing (e.g. small gene panels) to more comprehensive sequencing (WES or WGS)); the date of the most recent consideration by MSAC; the HTA approach used (i.e. CUC Proforma, investigative services, or codependent technologies); the type of economic evaluation relied on by MSAC; the key measure(s) of cost-effectiveness relied on by MSAC; MSAC’s assessment of the net costs to government likely to be associated with public funding for the proposed test; MSAC’s overall judgement of value; and whether the application was supported or not supported by MSAC. All monetary values are expressed in Australian dollars and reflect the year in which each assessment was undertaken. Dollar values have not been converted to a single year given the short timeframe of analysis (2016 to 2020). In addition, key challenges faced by MSAC during their appraisal were extracted from the PSDs and organised by theme.

A narrative synthesis was performed to compare the general features and evaluation components of the applications. The authors then individually and collectively reflected on the data to describe how MSAC’s view of its information needs has evolved over time in response to the diversity of applications assessed. The quality of the individual applications has not been appraised as part of this review as all contracted assessments and PSDs are prepared according to quality standards monitored by the Australian Department of Health. Where key pieces of information were missing from a PSD, this information was requested from the Department of Health and provided to us when appropriate to do so.

## Results

### Characteristics of selected studies

A total of ten applications, considered by MSAC between March 2016 and November 2020, met our study inclusion criteria (see Table 1). All published documents associated with an application were reviewed for relevant information, in particular: one application (breast and ovarian cancer) has two PSDs, an original application (1411), and a resubmission (1411.1), and the most recent assessment for non-invasive prenatal testing (NIPT) was application 1492, but earlier requests for this service had been initiated (1458 and 1461). Each included application was categorised according to the main purpose of testing the initial target population(s): three applications have been for ‘identification of congenital anomaly syndrome’ (childhood syndromes, NIPT, and foetal structural anomalies); two applications have been for ‘genetic testing for risk of cancer’ (breast and ovarian cancer, and colorectal and endometrial cancer risk); two applications for ‘molecular diagnosis of genetic disorder’ (Alport syndrome, and cardiac arrhythmias); and one application for each of ‘genetic risk assessment’ (familial hypercholesterolaemia), ‘pharmacogenomics’ (Ovarian BRCA1/2 for olaparib), and ‘carrier screening’ (carrier testing for cystic fibrosis (CF), spinal muscular atrophy (SMA), and fragile X syndrome (FXS)). The type of testing varied across the ten applications, from targeted gene panels to whole-exome sequencing and genome-wide microarray.

**Table 1:** Description of recent^1^ requests to MSAC for public funding of genetic or genomic testing for suspected heritable conditions

Appl number(s) (reference)	Title	Purpose of testing	Initial target population(s) to be tested	Scale of testing proposed	Date considered^2^
1411 1411.1 (MSAC 2016b)	Genetic testing for hereditary mutations predisposing to cancer (breast and/or ovarian)	Genetic testing for risk of cancer	Individual with breast or ovarian cancer	Sequencing of at least the *BRCA1* and *BRCA2* genes	Mar 2016
1449 (MSAC 2018a)	Genetic testing for Alport syndrome	Molecular diagnosis of genetic disorder	Individual clinically suspected to have Alport syndrome	Targeted whole-exome sequencing of the *COL4A3, COL4A4* and *COL4A5* genes	Mar 2018
1476 (MSAC 2019a)	Genetic testing of childhood syndromes	Identification of congenital anomaly syndrome	Child (10 years or younger) with suspected genetic syndrome due to dysmorphic facial appearance, one or more structural anomalies, intellectual disability or global developmental delay of at least moderate severity	Untargeted whole-exome sequencing	Aug 2019
1458 1461 1492 (MSAC 2019b)	Non-invasive prenatal testing (NIPT)	Identification of congenital anomaly syndrome	Any pregnant woman to detect foetal aneuploidy	Chromosome analysis for trisomy 21 (Down syndrome), trisomy 18 (Edward syndrome), trisomy 13 (Patau syndrome), and monosomy X (Turner syndrome)	Nov 2019
1504 (MSAC 2018b)	Heritable mutations which increase risk in colorectal and endometrial cancer	Genetic testing for risk of cancer	Individual with personal history of colorectal or endometrial cancer suggestive of hereditary basis, incl: juvenile polyposis syndrome, Peutz-Jeghers syndrome, hereditary mixed polyposis syndrome, suspected Lynch syndrome, familial adenomatous polyposis, or MUTYH-associated polyposis	Testing of the following genes: *APC*, *SMAD4*, *BMPR1A*, *MLH1*, *MSH2*, *MSH6*, *PMS2*, *STK11*, *GREM1*, *MUTYH*, EPCAM* [*deletions associated with epigenic silencing of *MSH2*]	July 2018
1533 (MSAC 2019c)	Testing for pregnancies with major foetal structural abnormalities detected by ultrasound	Identification of congenital anomaly syndrome	A pregnant woman where antenatal ultrasound has detected major foetal structural abnormalities	Genome-wide micro-array	Nov 2019
1534 (MSAC 2019d)	Familial hypercholesterolaemia	Genetic risk assessment	Individuals with elevated levels of LDL-cholesterol who are clinically suspected as having familial hypercholesterolaemia	Genetic testing of at least the following genes: *LDLR*, *APOB*, PCSK9	Mar 2019
1554 (MSAC 2019e)	Germline or somatic testing for BRCA1/2 to determine eligibility for olaparib	Pharmacogenomics	Individuals with newly diagnosed, advanced (FIGO stage III–IV), high-grade epithelial ovarian, fallopian tube, or primary peritoneal cancer, to determine PBS eligibility for olaparib	Blood and tumour tissue testing to detect germline or somatic pathogenic variants in the *BRCA1* or *BRCA2* genes	Jul 2020
1573 (MSAC 2020a)	Reproductive carrier testing for cystic fibrosis (CF), spinal muscular atrophy (SMA), and fragile X syndrome (FXS)	Carrier screening	Individual and/or their reproductive partner planning a pregnancy	For CF: *MALDI-TOF*, allele specific amplification, Sanger sequencing or NGS for CFTR variants, and for SMA: MLPA probes or qPCR for *SMN1*, and for FXS: fragment analysis (primed PCR to detect triplet repeat expansion)	Jul 2020
1598 (MSAC 2020b)	Genetic testing for diagnosis of inheritable cardiac rhythm disorders	Molecular diagnosis of genetic disorder	Individuals with clinical suspicion of inherited cardiac arrhythmia or channelopathy	Genetic testing (including copy number variation) of at least the following genes: *KCNQ1*, *KCNH2*, *SCN5A*, *KCNE1*, *KCNE2*, *KCNJ2*, *CACNA1C*, *RYR2*, *CASQ2*, *CAV3*, *SCN4B*, *AKAP9*, *SNTA1*, *KCNJ5*, *ALG10, CALM1*, *CALM2*, *ANK2*, *TECRL*, and *TRDN*	Nov 2020

*CFTR*, cystic fibrosis transmembrane conductance regulator protein; *FIGO*, the International Federation of Gynecology and Obstetrics; *MLPA*, multiplex ligation-dependent probe amplification; *NGS*, next-generation sequencing; *PBS*, pharmaceutical benefits scheme; *PCR,* polymerase chain reaction; *qPCR*, quantitative polymerase chain reaction

^1^ ‘recent’ is defined as applications considered by MSAC at their March 2016 meeting or later and where a public summary document (PSD) was available at the time of analysis

^2^Date of consideration is the most recent consideration by MSAC. Some applications had more than one consideration by MSAC and may have more than one PSD

Table 2 presents key aspects of the HTA approach taken for the ten applications. All but one of the applications (NIPT) was ultimately supported by MSAC for public funding. Six applications used the CUC Proforma to frame the HTA, three applications used the investigative services approach, and one used the codependent technologies approach. A cost-utility analysis (CUA) was undertaken for four applications, and a cost-effectiveness analysis (CEA) was undertaken for five applications. In one application, the economic analysis was deemed by MSAC to be unreliable (Alport syndrome).

**Table 2:** Key aspects relied on by MSAC to make judgements of value

Appl. no	Short title	HTA approach	Type of economic analysis relied on by MSAC	Key measure(s) of cost-effectiveness relied on by MSAC	MSAC assessment of net costs to government	MSAC’s judgement of value *(supported/not supported for funding)*
1411	Breast and ovarian cancer risk	CUC Proforma—pilot	CUA	• $18,283 per QALY • $32,000 per breast or ovarian cancer event avoided	• $5.05 M to $7.01 M per year • Assumes 10% of breast/ovarian cancer cases will be eligible for testing	• Clinical utility: Change in cancer risk management for index case Change in cancer risk management for relatives *Supported*
1449	Alport Syndrome	CUC Proforma	none (CUA in assessment report deemed unreliable)	• n/a	• $2.45 M to $1.37 M per year^1^ • Well characterised phenotype with a low risk of use outside the intended population	• Clinical utility Avoidance of renal biopsy Prognostic information • Reproductive confidence *Supported*
1476	Childhood syndromes	CUC Proforma	CUA	• $7254 per QALY	• $10.27 M to $9.52 M per year^2^ • Includes initial WEA, re-analysis, and cascade testing but excludes downstream savings as difficult to quantify	• Clinical utility: Avoidance of diagnostic testing for index case Avoidance of unnecessary treatments • Reproductive confidence *Supported*
1492	NIPT	Investigative services	CEA	• $510,769 per additional trisomy detected	• $100 M per year^3^ • Assumes NIPT would be used in addition to biochemical testing and antenatal ultrasound in ~ 300,000 pregnancies per year	• Reproductive confidence *Not supported*
1504	Colorectal and endometrial cancer risk	CUC Proforma	CEA	• $9762 per additional mutation detected (for Lynch syndrome index case) • $5691 per mutation identified (for Familial Polyposis index case)	• $3.27 M to $3.48 M per year^3^ • Does not include expected mix of downstream costs and savings associated with cancer surveillance, risk-reducing surgeries, or cancer treatment	• Clinical utility: Change in cancer risk management for index case Change in cancer risk management for relatives *Supported*
1533	Foetal structural abnormalities	Investigative services	CEA	• $5258 per pathogenic CNV detected • Cost per complex birth avoided – testing dominates	• $1.71 M to $1.74 M per year^3^ • Use outside intended population is expected to be low as amniocentesis and CVS are unlikely to be used inappropriately	• Clinical utility: Guiding management of ongoing pregnancy *Supported*
1534	Familial hyper-cholesterolaemia	CUC Proforma	CUA	• $24,907 per QALY (for testing affected individuals plus first- and second-degree relatives)	• $0.54 M to $0.62 M per year^3^ • Assumes uptake is based on current rates of under-diagnosis of Familial Hypercholesterolaemia	• Clinical utility: Change in risk management for biological relatives Access to PBS-funded pharmacotherapy for index cases *Supported*
1554	Ovarian cancer BRCA1/2 for olaparib	Codependent technologies	CUA	• Cost per QALY^4^	• Net cost to MBS^4^ • For extending existing BRCA1/2 germline testing to allow somatic BRCA1/2 testing to determine patient access to first-line olaparib	• Clinical utility: Access to PBS-funded pharmacotherapy for index cases *Supported*
1573	Carrier testing for CF, SMA and FXS	Investigative services	Revised CEA	• Cost per carrier couple detected – Carrier testing dominates	• $34.90 M to $35.23 M per year^3^ • Plus possible savings to the PBS of $2.67 M to $17.54 M per year	• Reproductive confidence: For clinically warranted testing of women early in pregnancy or intending to become pregnant, and their reproductive partners *Supported*
1598	Cardiac arrhythmias and channelopathies	CUC Proforma	CEA	• $4721 per positive genotype^5^ for affected individuals • Testing dominates for affected individuals plus first-degree relatives, and for affected individuals plus first-and second-degree relatives	• $0.56 M to $1.25 M per year^3^ • Long-term impacts of services associated with changed prevention and surveillance are uncertain	• Clinical utility: Change in risk management for biological relatives • Reproductive confidence: Reproductive partner testing for genes with autosomal recessive inheritance *Supported*

*CEA*, cost-effectiveness analysis; *CF*, cystic fibrosis; *CNV*, copy number variant; *CUA*, cost-utility analysis; *CVS*, chorionic villus sampling; *FXS*, fragile X syndrome; *MBS*, Medicare Benefits Schedule; *MSAC*, Medical Services Advisory Committee; *NIPT*, non-invasive prenatal testing; *PBAC*, Pharmaceutical Benefits Advisory Committee; *PBS*, pharmaceutical benefits scheme; *QALY*, quality-adjusted life year; *SMA*, spinal muscular atrophy; *WEA*, whole-exome analysis

^1^Net change in healthcare costs, including patient contributions

^2^Total cost to MBS and patients

^3^Cost to MBS

^4^Values redacted in PSD

^5^Positive genotype defined as presence of pathogenic or likely pathogenic variant

Where the base-case incremental cost-effectiveness ratio (ICER) has been published for the CUAs, it ranged from $7,254 per quality-adjusted life year (QALY) to $24,907/QALY. Assessments that relied on a CEA used a variety of units to express cost-effectiveness: cost per pathogenic variant detected (specific mutations, copy number variants, or presence of trisomy), cost per complex birth avoided, or cost per carrier couple detected. Although these ICERs cannot be directly compared because of the different units used, it was noted that the requests where testing was dominant or yielded an ICER less than $10,000 per outcome were ultimately supported by MSAC, whereas the testing that was not supported by MSAC (NIPT) was associated with an ICER of more than $500,000 per outcome. For the eight applications that were supported by MSAC and for which the government budget impacts are reported, the financial estimates ranged from less than $1 M per annum to less than $10 M per annum. For the one assessment that was not supported by MSAC (NIPT), the financial estimate was in the order of $100 M per annum.

For eight of the applications MSAC’s judgement of value relied primarily on clinical utility to affected individuals and/or their family members (typically through avoidance of further diagnostic tests, changes in disease risk management, or the avoidance of unnecessary treatments), and in the remaining two applications, it relied on reproductive confidence for the tested individuals.

**Fig. 1:**
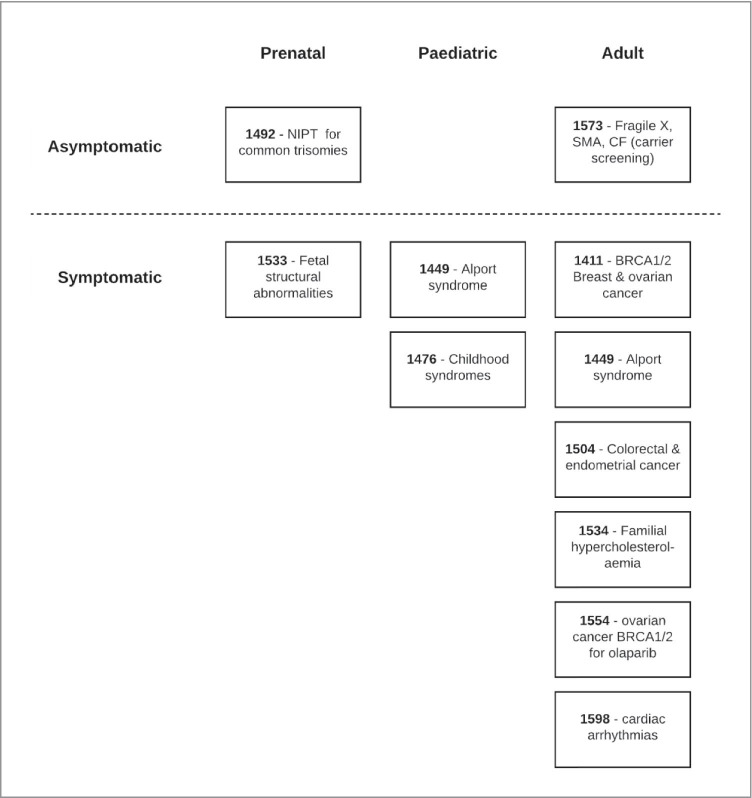
MSAC applications by features of the primary population for testing. Note: Application 1449 appears twice as testing of affected individuals can be undertaken in children or adults. Abbreviations: CF, cystic fibrosis; CUC, clinical utility card; Fragile X, fragile X syndrome; MSAC, Medical Services Advisory Committee; NIPT, non-invasive prenatal testing; SMA, spinal muscular atrophy

**Fig. 2:**
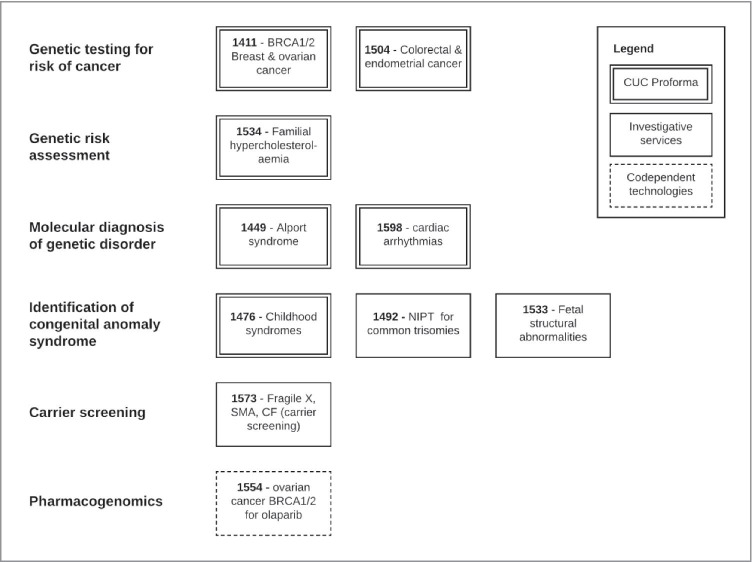
MSAC applications by purpose of testing and HTA approach used. Abbreviations: CF, cystic fibrosis; CUC, clinical utility card; Fragile X, fragile X syndrome; MSAC, Medical Services Advisory Committee; NIPT, non-invasive prenatal testing; SMA, spinal muscular atrophy

Figure 1 presents the applications arranged by key features of the tested populations: symptomatic or asymptomatic and broad age group (prenatal, paediatric, adult). Figure 2 shows the applications organised by purpose of testing and type of HTA approach used. The figures illustrate the diversity of populations and testing purpose reflected in the ten applications. Eight out of ten applications were for initial testing in populations that were symptomatic (although cascade testing could have been in asymptomatic family members), and seven applications were for initial testing in adults (Fig. 1). The CUC Proforma approach was used for applications concerned with genetic testing for risk of cancer, genetic risk assessment, or molecular diagnosis of a genetic disorder. Applications for the purpose of identifying a congenital anomaly syndrome used either the CUC Proforma or the investigative services approach. The single application concerned with carrier screening used the investigative services approach, and the single application for testing for a pharmacogenomics purpose used the codependent technologies approach.

### Synthesis of findings

**Fig. 3:**
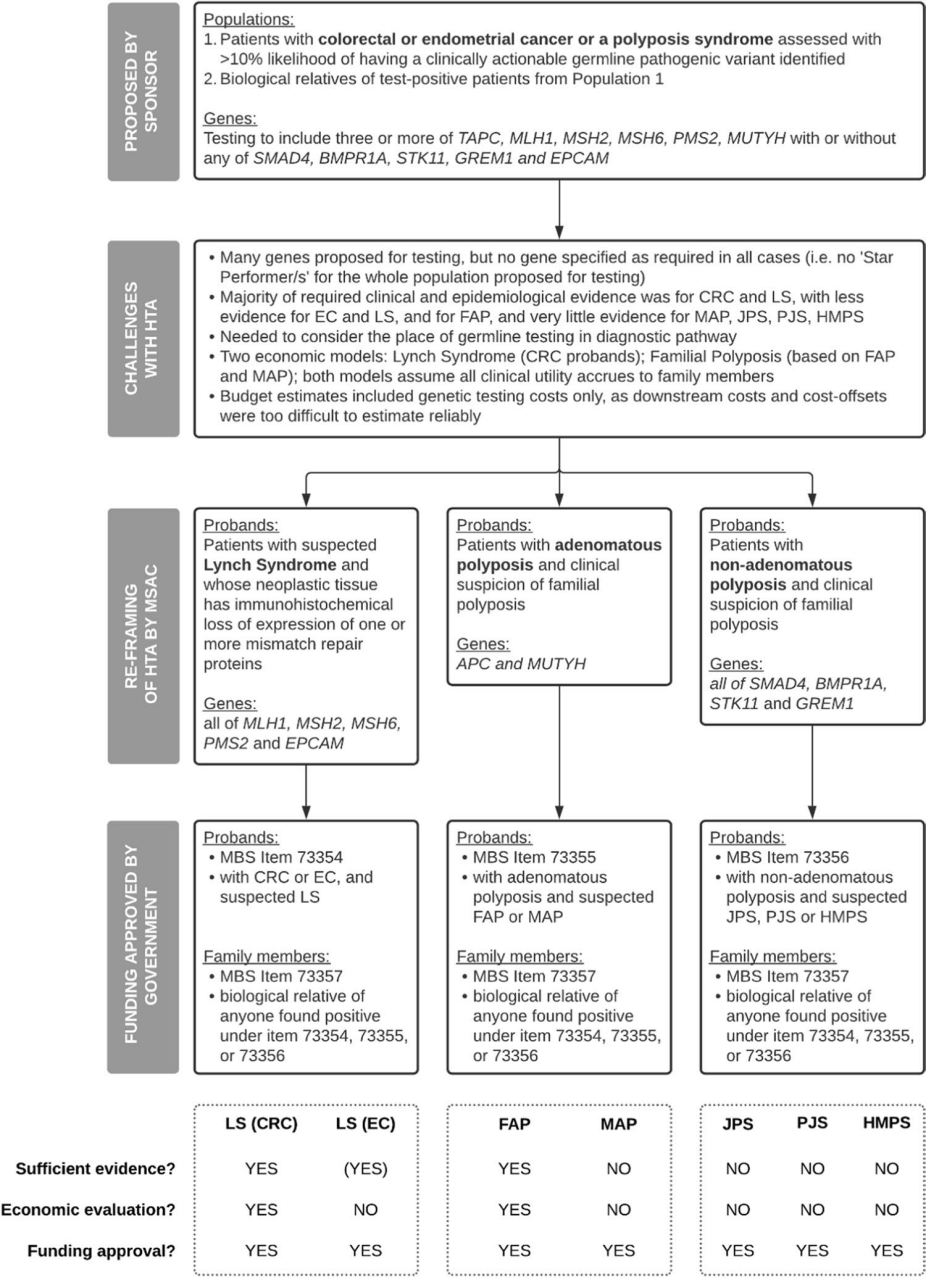
Example showing how evidence and economic evaluations from one population were accepted by MSAC as the basis for funding approval for clinically related populations with less evidence. Abbreviations and definitions: CRC, colorectal cancer; EC, endometrial cancer; FAP, familial adenomatous polyposis; HMPS, hereditary mixed polyposis syndrome; JPS, juvenile polyposis syndrome; LS, Lynch syndrome; MAP, MUTYH-associated polyposis; MBS, Medicare Benefits Schedule; MSAC, Medical Services Advisory Committee; PJS, Peutz-Jeghers syndrome; star performer, defined by MSAC as high penetrance, actionable gene(s) within a disease area that are likely to have the clearest evidence of clinical utility

The lessons learnt by MSAC during the assessment of the ten applications are summarised by theme in Table 3. These themes reflect the HTA domains across the CUC Proforma and the MSAC guidelines for investigative services and codependent technologies. Specific aspects that were challenging for assessment are listed, together with the approach taken by MSAC to address these challenges. A summary of the approaches adopted in assessing application 1504 is presented as Fig. 3, to illustrate some of the key themes. These themes are described in more detail below.

**Table 3:** Lessons learnt during the assessment of relevant MSAC applications

Theme Specific aspect	Why this aspect was a challenge for HTA	How challenges have been addressed by MSAC (to date)	Examples
Health condition			
Proposed population too broad and heterogenous	Difficult to establish link between specific genotype and disease/risk (clinical validity), no ‘Star Performer’ gene(s)	Redefined (sub)populations for testing based on pathological and clinical characteristics (see Fig. 3)	1476 1504
Less common conditions	Limited clinical and/or epidemiological evidence as required by MSAC approach	Used evidence-based funding recommendations for clinically related populations to support funding for health conditions with less evidence (see Fig. 3)	1411 1504
Natural history of condition not known or not specified	Difficult to define health outcomes for individuals who do not receive testing	Shortened time horizon to reduce uncertainty (e.g. focus on avoidance of diagnostic odyssey)	1449
Pathways of care			
Insufficient consideration of how and when an individual would be considered for testing	Unclear if proposed test would replace or be used in addition to current tests	Sought input from a wider range of stakeholders, and/or referred to relevant, current evidence-based guidelines to develop funding advice	1492 1504
	Place in diagnostic pathway unclear, especially with regard to use of triaging tests such as tumour histopathology		
Pre-test probability of a positive result set at 10% by MSAC	Threshold challenged by Sponsors, due to perceived difficulties in quantifying this probability for many health conditions	Has not been a barrier to funding approvals for testing in affected individuals, in most cases the pre-test probability has been > 10%, and funding has been approved in specific instances where it has been < 10% but there is a high unmet need and high clinical utility	1411 1449 1504 1534 1573 1598
Insufficient consideration of who would request the test (referral for testing)	Broadening the type of health professionals who can request the test might improve access (see below) but might also lower diagnostic yield	Balance between facilitating access and optimising diagnostic yield considered on a case-by-case basis, and type of health professionals specified in the MBS item	All
Technology			
Test performance	Analytic validation against a reference standard is not always possible	Assume 100% sensitivity and specificity	All
	Different types of testing methodologies (see below) associated with a different diagnostic yield	Engagement with stakeholders and experts to identify the testing methodology most likely to be used in practice in Australia, and the relative clinical importance (by condition) of different diagnostic yields	1534 1573
Type and range of proposed molecular techniques	Wide range of different techniques used in different combinations, with rapid evolution of new techniques	Assumed most efficient testing approach will be used, so funding approval (generally) does not specify technique(s)	1554
	Potential for incidental findings is increased with the use of WES and WGS (see below)	MBS item only specifies WES or WGS if absolutely required	1476
Rapid emergence of knowledge regarding pathogenic variants	Not feasible (evidence or resources required to undertake HTA) to re-assess a test every time a new variant is identified	Developed MBS item for re-interrogation of whole exome or genome sequence data, and circumstances when this MBS item should apply	1476
Effectiveness			
Clinical utility	Insufficient information regarding the impact of test results on clinical management	Relied on clinical assumptions if appropriate, or if too uncertain shortened time frame of analysis to period before and immediately after the test	1449 1534
	Insufficient information regarding how change in clinical management impacts health outcomes		
‘Star Performer’ genes	Concept was useful for targeted gene panels but was not feasible/appropriate for large gene panels, chromosome analysis, WES or WGS	Relied on management of phenotype (irrespective of specific genes) to inform assessment	1449 1476 1492 1533 1598
Diagnostic yield	Varies based on patient selection and not always available for the proposed population for testing	Engagement with stakeholders and experts to identify the relative clinical importance, by condition, of different diagnostic yields Redefined the population for testing to align with the available evidence, or assumed transferability of evidence from one population to another	1476
Non-health outcomes	Supporting restoration of ‘reproductive confidence’ as a measure of test impact, without placing a value on avoiding the birth of an affected child	Expressed value in terms of diagnostic yield to avoid any perception that judgements were being made regarding the nature of reproductive decisions made	1476 1492 1533
		Developed MBS items for Reproductive partner testing	1476
	How to value a diagnosis that has no (immediate) clinical utility	Accepted that positive test results can allow access to educational or disability support services and have the potential to become actionable in the future, but only made a qualitative judgement of these benefits, given that MSAC does not take a societal perspective	1476
Safety			
Harms associated with test results	Potential to report ‘off-target’ mutations and variants of unknown significance, at initial sequencing and on re-interrogation	Assumed to be appropriately addressed via genetic counselling (see below) and quality programs in laboratories ensuring only pathogenic mutations are issued on molecular pathology reports	1476 1533
	Identification of disorders with no treatments or prevention interventions		
Ethics and equity			
Funding for pre- and post-test genetic counselling, for probands and for biological relatives	Recognised as integral to patient-centred care, but not coverable by the MBS	Assumed to be delivered by the clinician providing care, and included as a health system cost in the economic evaluations	All
Equitable access for all eligible individuals	Access will be limited if only clinical geneticists can request a test	Balance between equity and appropriateness of testing assessed by MSAC on a case-by-case basis	All
Cost effectiveness			
Cascade testing	In circumstances where only biological relatives experience benefits from testing, the economic model still needs to include testing of probands	Concept of ‘co-production of utility’ was implemented, which informs the structure of economic models, and allows exploration of cost-effectiveness in first-, second-, and third-degree relatives	1534 1598
Quality of life	Limited evidence of impact of genetic testing on QoL	CUA attempted, but MSAC relied on CEA if the quality of life data were too uncertain, or, an evidence-based economic evaluation in one population was used to inform the decision for a clinically similar condition with less evidence (see Fig. 3)	1411 1476 1492 1504
	Limited evidence for utility weights for individuals with health conditions other than cancer or cardiovascular disease		
Comparing ICERs	Difficult to directly compare cost-effectiveness across different tests when ICERs expressed in different units	Where possible, additional ICERs have been derived based on a relevant measure of diagnostic yield	1492 1504 1533 1573 1598
Budget impact			
Number of individuals likely to be eligible for testing	Number of affected individuals who meet the criteria for testing will be larger than the number of individuals with the specific genetic variants of interest	Sought additional clinical input regarding the prevalence of individuals with the characteristics that would make them eligible for the proposed test	1449 1598
	Potential for use in unintended populations	Restrictions on eligibility for testing and on the test referrer	1476
Downstream costs and cost-offsets	Limited evidence on cost consequences of testing	If highly uncertain then budget impact based solely on costs of testing	1504 1534
Legal issues			
Potential to detect non-paternity, consanguinity or incest	Outside the usual range of matters considered by MSAC, and represent broader policy issues that cannot be addressed via the MBS	Not resolved	Potentially all
Data storage and privacy			
Possible forensic uses of sequence data			
Implementation			
Cascade testing	Limited evidence of rate of uptake of cascade testing in biological relatives	Assumptions made regarding ‘average’ uptake in families, and alternative scenarios tested in sensitivity analyses	All except 1411
Uptake of risk-reducing strategies in probands and biological relatives	Limited evidence of rates of uptake of different risk-reducing strategies (and age at uptake)	Assumptions made regarding uptake, which are then tested in sensitivity analyses	All except 1411
Health system efficiency	Tension between broad access to cost-ineffective testing versus access to cost-effective testing for individuals at highest risk	Not resolved	1492

*CEA*, cost-effectiveness analysis; clinical utility; the net health benefit/harm derived from an investigative health technology across all those tested (including true positives, false positives, true negatives, and false negatives); *CUA*, cost-utility analysis; diagnostic odyssey, the series of investigative services a patients undergoes until they receive a diagnosis; *HTA*, health technology assessment; *ICER*, incremental cost-effectiveness ratio; *MBS*, Medicare Benefits Schedule; *MSAC*, Medical Services Advisory Committee; *QOL*, quality of life; star performer; the actionable gene(s) for which the strongest clinical utility and/or cost-effectiveness argument is likely to apply for an affected individual; trio testing, when parents are tested at the same time a child is tested; WES, whole-exome sequencing; *WGS*, whole-genome sequencing

### Health condition

Clinical validity (the link between a genotype and a disease or disease risk) was easier to assess in circumstances with a limited number of involved genes and a specific phenotype (e.g. Alport syndrome), but harder to assess when there were multiple involved genes and a non-specific phenotype (e.g. Childhood syndromes), or when the condition was rare (e.g. Peutz-Jeghers syndrome). For some applications, MSAC redefined the populations to be assessed and used evidence of effectiveness, safety, and cost-effectiveness in one population to infer effectiveness, safety, and cost-effectiveness in a clinically related population. For example, application 1411 was underpinned by the evidence linking BRCA1/2 testing in breast cancer probands, and this was used to support public funding for BRCA1/2 testing in breast cancer probands and ovarian cancer probands. In application 1504, a heterogenous population of individuals with suspected heritable colorectal or endometrial cancer was redefined as three patient groups which better reflected the linkage between genotype and condition (see Fig. 3). Evidence of the effectiveness, safety, and cost-effectiveness of genetic testing for Lynch syndrome in colorectal cancer probands was then used to inform public funding decisions for genetic testing for Lynch syndrome in endometrial cancer probands and genetic testing for five familial polyposis syndromes.

### Pathways of care

Many applications focused on the genetics of the condition(s) in question and the technical performance of the genetic/genomic test (see below), without sufficient consideration of how and when an individual would be considered for testing (i.e. prior testing), the type of health professional who would request the test, and whether the proposed test would replace or be used in addition to current testing. MSAC typically referred to evidence-based clinical practice guidelines to better understand the likely pathways of care, with and without the proposed test (e.g. 1504, 1598).

The most appropriate place in care for genetic/genomic testing was most difficult to define when the testing was proposed for asymptomatic individuals (see Fig. 1): prenatal screening (1492) and carrier screening for rare diseases (1573). The challenges included different views held by different clinical specialities; established use of biochemical methods with acceptable accuracy and cost; established uptake of genomic testing in the private sector; and different funding responsibilities between the federal and state governments. Similar challenges have been discussed by Cornel et al. (2021), who describe how innovations in sequencing technology are challenging established principles for screening.

As noted in the “Introduction”, MSAC’s preference for limiting germline testing to populations with a higher likelihood of an actionable result is reflected in the requirement for a > 10% pre-test probability that an individual will harbour pathogenic variants in one or more specified genes. Although the selection of this threshold was questioned by sponsors, it has been very helpful in defining the proposed population for testing by more explicitly articulating the clinical presentation and findings from prior tests.

### Technology

MSAC found that analytic validity was difficult to ascertain when multiple molecular techniques can be used in different combinations, and the studies of test performance do not always describe the techniques used. To address this, MSAC assessed the available evidence and then tended to assume 100% sensitivity and specificity for the corresponding economic evaluations. Over time, MSAC has placed more focus on the diagnostic yield of a test, the applicability of the available evidence to the proposed use of the test in Australian clinical practice, and the relative clinical importance of different diagnostic yields.

It is well-known that genetic/genomic testing is undergoing a high rate of technological innovation with a concurrent rapid increase in the knowledge base of genes involved in disease. The dynamic features of this class of health technology are challenging for HTA, which is essentially a static assessment (i.e. at a point in the product life cycle). MSAC has sought to address the first aspect, rapid technological innovation, by make funding approvals largely agnostic to the molecular technique(s) used, in order to ‘future proof’ the MBS items as much as possible. The second point has been addressed in two ways: (i) limiting the evidence assessment requirements to a small number of genes per heritable condition, but then recommending funding for a broader list of genes for that condition (i.e. the ‘Star performer gene’ concept), and (ii) developing MBS items for future re-interrogation of sequencing data, where appropriate.

### Effectiveness

As noted above, applications tended to focus on the technical performance of a test, and in many instances, there was limited evidence regarding the impact of test results on clinical management and/or health outcomes. This made it challenging to establish clinical utility for a test, either directly or indirectly, through the use of linked evidence. Rather than reject requests for funding on the basis of a failure to demonstrate clinical utility, MSAC sought to base its advice on assumptions regarding the impact of test results. If these assumptions were highly uncertain, MSAC reduced the uncertainty by shortening the time horizon of the analysis to the period before and immediately after the test (1449, 1534). Ultimately, the demonstration of clinical utility was the basis of decision-making for eight of the nine applications that received funding approval. In two of these eight applications (1534, 1598), the clinical utility accrued entirely to the biological relatives of the affected individuals/probands.

MSAC has attempted to place a value on two of the potential non-health outcomes of genetic or genomic testing: restoration of reproductive confidence and providing a diagnosis that has no immediate clinical utility. In five applications (1449, 1476, 1492, 1573, 1598) reproductive confidence was judged by MSAC to be an important measure of test effectiveness. In some assessments, attempts were made to predict the reproductive choices that would be made with and without the test results, but MSAC found these predictions to be highly uncertain. Moreover, MSAC was acutely aware that the outcomes associated with reproductive decisions (e.g. using in vitro fertilisation methods, pregnancy termination, giving birth to an affected child) are valued differently by different individuals. Consequently, MSAC elected to express value in terms of diagnostic yield, such as the number of additional trisomies detected (1492) or the number of carrier couples detected (1573). MSAC acknowledged that both negative and positive test results can inform reproductive decisions, but expressing cost-effectiveness in terms of the cost per ‘informed reproductive decision’ would produce ICERs that are uninformative for MSAC as they would simply equate to the cost of the test.

The value of providing a diagnosis with no immediate clinical utility is exemplified by application 1476 and WES for the diagnosis of childhood syndromes. In its consideration, MSAC acknowledged that there was value in receiving a confirmed molecular diagnosis as, in addition to restoring reproductive confidence, it likely ended the diagnostic odyssey for the family and could allow access to educational or disability support services. Evidence consistent with this conclusion was later published: a discrete choice experiment found that members of the Australian public highly valued the non-clinical components of genomic sequencing in complex paediatric neurological disorders (Goranitis et al 2021).

### Safety

As with all investigative services assessed by MSAC, each genetic or genomic test was evaluated for safety in terms of the methods used to collect the biological specimen for testing (e.g. blood sample, tissue biopsy). In addition, MSAC recognised that genetic and genomic testing have the potential to cause psychological harm to the individuals undergoing testing. This harm can arise through the identification of ‘off target’ mutations or non-actionable genetic variants (variants of unknown significance, or variants with no known treatment). Harms can also arise as a consequence of detecting non-paternity, consanguinity, or incest (see “Legal issues” below).

The importance of measuring the impact of genetic testing using patient-centred outcomes has been recognised internationally for many years, but there are very few high-quality studies that describe these impacts (Phillips et al. 2017). Psychological impacts that are measurable using validated tools could be included in any MSAC application, but to date, none of the applications considered by MSAC has incorporated this potential impact of testing.

### Ethics and equity

A recurring challenge across the MSAC assessments was how best to capture the requirements for pre- and post-test genetic counselling. Whilst MSAC accept that genetic counselling is an integral part of patient-centred care, it is not coverable as a stand-alone service on the MBS (due to legislative constraints). This challenge was managed by MSAC in a ‘technical’ sense by assuming that the clinician referring the individual for testing would provide the required counselling and by including the costs for delivering genetic counselling within the economic evaluations.

Ensuring equity of access to safe, effective, and cost-effective care is a key principle that guides the development of all MSAC advice. For each application, MSAC explicitly considered which health professionals would be eligible to request an MBS-funded test, recognising the limited number of clinical geneticists in Australia. In doing this, MSAC sought to find a balance between increasing the access to testing (by broadening the range of requestors) whilst maintaining diagnostic yield (i.e. effectiveness).

### Cost-effectiveness

As reported by Phillips et al. (2018), key challenges for MSAC in assessing economic evaluations have fallen into three domains of complex model structures (to capture the multiple pathways of use of testing), the measurement of costs and outcomes, and a lack of good quality data. When there was a high level of uncertainty regarding the downstream consequences of testing due to a paucity of reliable evidence, MSAC tended to rely on a CEA (instead of its preference for a CUA, e.g. 1449,1534) and/or a shorter time horizon for the assessment (namely, a focus on the pathway of care prior to and immediately after testing; e.g. 1504, 1598). In one application (1449), MSAC essentially relied on a cost-consequence analysis and noted that ‘while the evidence base for genetic testing [in Alport syndrome] was limited, there was acceptable evidence of clinical safety and effectiveness, and the financial impact of funding was likely to be low in the context of this rare disease with a well-characterised phenotype’.

In other circumstances (1504, 1598) there was so little evidence that even an economic evaluation with a short time horizon could not be reliably developed. In these situations, demonstration of cost-effectiveness in a clinically similar population was accepted by MSAC as being broadly applicable to the population with insufficient evidence. This is illustrated in Fig. 3: evidence-based cost-effectiveness of testing probands with colorectal cancer and suspected Lynch syndrome was used to support funding for probands with endometrial cancer and suspected Lynch syndrome, and evidence-based cost-effectiveness for testing individuals with suspected familial adenomatous polyposis (FAP) was used to support funding for individuals suspected as having MUTYH-associated polyposis (MAP), juvenile polyposis syndrome (JPS), Peutz-Jeghers syndrome (PJS), or hereditary mixed polyposis syndrome (HMPS).

As noted above, the demonstration of the clinical utility of testing for probands and/or biological relatives was the primary basis for MSAC decision-making: for two applications, clinical utility accrued entirely to the biological relatives (1534, 1598). Applying the concept of co-production of health-related utility was important for all these applications, especially the latter two, as it provided a consistent framework for structuring and reporting the economic evaluations. This approach to the economic evaluations also framed marginal analyses to explore the relative cost-effectiveness of extending testing beyond first-degree relatives to second- and third-degree relatives (e.g. 1534).

The challenges encountered by MSAC in assessing the costs and cost-effectiveness of genetic and genomic testing are similar to those reported by others. A systematic review of the health economic evidence for genetic and genomic testing (across a range of clinical purposes similar to those described herein) found that few studies reported data transparently and/or did not state which components were included in cost estimates (Schwarze et al. 2018). Like MSAC, these authors found there was a very limited body of evidence regarding the impact of genetic/genomic tests on intermediate or final health outcomes.

### Budget impact

A common challenge with establishing budget impact estimates was determining the number of individuals likely to be eligible for testing. Many applications provided information on the prevalence of specific genetic variants, but provided little or no information on the (larger) number of individuals *suspected* as having those variants, or the extent of testing uptake in biological relatives. MSAC addressed these issues in a technical sense (by testing assumptions regarding eligible population size in sensitivity analyses) and via implementation (i.e. proposing MBS item descriptors with clear criteria for testing).

In applications where there was high degree of uncertainty regarding downstream costs and consequences of testing, MSAC relied on assessments of budget impact that were limited to the costs of delivering testing (i.e. the genetic/genomic test itself, plus any other tests or services required to deliver the test; e.g. 1504, 1534).

### Legal issues

In addition to the ethical issues noted above, MSAC has acknowledged legal issues that might arise as consequence of genomic testing, including data storage and privacy, possible forensic uses of sequencing data, and the potential to detect non-paternity, consanguinity, or incest. These matters have not been addressed by MSAC as they represent broader policy issues that fall outside the remit of the committee.

### Implementation

For each application, MSAC considered the likely uptake of cascade testing and the likely uptake of prevention and treatment strategies in probands and biological relatives. Direct evidence to inform these aspects of the assessment was seldom available, necessitating the use of reasonable assumptions to inform estimates of cost-effectiveness and budget impact. The validity of these assumptions is usually tested following implementation of the corresponding MBS items, via a comparison of ‘predicted’ versus ‘actual’ utilisation of the items.

Uncertainty regarding the likely place of testing in clinical care also presented challenges for implementation. For the majority of applications, the appropriate place in care for the proposed test could be determined by triangulating clinical advice, recommendations from clinical practice guidelines, and existing subsidies for tests and treatments. But for application 1492 (NIPT), the lack of clarity regarding how the test would be implemented in the Australian health system contributed to this test not being supported for public funding. Application 1492 also presented issues regarding health system efficiency, as there was a (unresolved) tension between broad access to cost-ineffective testing versus targeted access to costs-effective testing for individuals at highest risk.

## Value assessment frameworks for genetic and genomic testing

As shown in Fig. 2, each of the ten applications for testing for heritable conditions have used one of three available MSAC approaches. Except for the single use of the codependent technology approach for the pharmacogenomic application (1554), there is no clear pattern of use for the CUC Proforma versus the Investigative Services approach. The current plan is that key concepts from the CUC Proforma as they relate to testing for heritable conditions will be incorporated within the technical guidance for investigative services, namely, the inclusion and implications of cascade testing (including coproduction of utility), and the consideration of utility other than clinical utility.

## Discussion

The aim of this paper was to review MSAC’s experience in assessing genomic and genetic tests for heritable conditions and to share current thinking regarding the need for further evolution of MSAC’s approach to the assessment of genetic and genomic testing. The ten applications included in this review cover a range of testing purposes: identification of congenital anomaly syndrome, genetic testing for risk of cancer, molecular diagnosis of genetic disorder, genetic risk assessment, pharmacogenomics, and carrier screening. The type of testing across these applications varied from targeted gene panels through to whole-exome sequencing and genome-wide microarray. Although efforts were made to adopt a pragmatic HTA approach a priori, none of these assessments ended up being straightforward. Challenges faced during the assessments included a lack of clarity regarding who would be tested and when and a lack of evidence for both the positive and negative impacts of testing. The uncertainty regarding the appropriate place of testing in relevant pathways of care and the clinical utility of testing lead to uncertainty in the estimations of cost-effectiveness and total budget impact. Nonetheless, despite these challenges, nine of these applications were ultimately supported by MSAC for public funding.

The approaches taken by MSAC to value genetic or genomic testing for heritable conditions are similar to assessment frameworks for ‘omic’ technologies used by HTA agencies around the world (Hoxhaj et al. 2020), including a focus on the link between genotype and disease, the evaluation of test performance, and the utility of testing. Many of the challenges faced by MSAC in assessing genetic and genomic tests are not unique to Australia. For example, a systematic review of economic evaluations of NGS-based tests in Canada found there were challenges related to inconsistent approaches to the conduct and reporting of economic analyses, the uncertainty related to evidence gaps, and the lack of incorporation of non-health outcomes into economic evaluations (Weymann et al. 2019). Whilst the first of these has not been an issue in the MSAC context (each application complies with MSAC guidelines which are consistent with international standards for reporting of HTA and health economic analyses; Husereau et al. 2013; Drummond et al. 2015), the lack of key evidence and difficulties in valuing non-health benefits have been consistent challenges for MSAC.

Although the concept of clinical utility is fundamental to how MSAC assesses the value of any investigative service, MSAC has acknowledged a number of positive and negative non-health impacts of genetic and genomic testing: providing or restoring reproductive confidence; facilitating access to diagnosis-dependent educational and/or disability support; identification of disorders for which there are no prevention or treatment interventions; detection of non-paternity, consanguinity, or incest; possible forensic uses of sequencing data; and issues around data storage and privacy. Where appropriate, MSAC sought to take these (potential) non-health outcomes into consideration when forming their overall judgement of value for a specific testing scenario. Many of these non-health impacts carry potentially significant ethical and social impacts, and the more comprehensive the sequencing, the greater the potential for ethical and social impacts. MSAC has acknowledged that it would be desirable for the societal value placed on the ‘value of knowing’ from genetic testing to be captured more formally in future assessments.

As noted above, MSAC has a strong preference for genetic or genomic testing that yields actionable results. But consumers and clinicians differ in what they view as ‘actionable’: for a patient, the benefits of genomic testing can also include knowledge generated by the test that is not clinically actionable, referred to as ‘personal utility’ (Bombard et al. 2013; Kohler et al. 2017). Whilst Australian patient preferences for health and non-health outcomes associated with genomic testing have been reported (e.g. Goranitis et al. 2020, Goranitis et al. 2021), MSAC does not yet have preferred methods for the elicitation of such preferences, or their incorporation into MSAC assessments.

A recent systematic review of studies that valued patient preferences for NGS outcomes identified a number of methodological and conceptual challenges in the elicitation of these preferences, which they concluded were related to the breadth and complexity of information associated with NGS-directed care (Regier et al. 2018). Others have argued for an expanded HTA value framework that includes elements such as reduction in uncertainty, the value of hope, and the value of knowing information that might guide treatment at a future date (Garrison et al. 2017). However, the methodology for incorporating these aspects of value into an HTA is an area of active research, and possible methods such as willingness-to-pay and cost–benefit analysis are generally not preferred by MSAC. A promising concept proposed by Mighton et al. (2020) is that personal utility and clinical utility lie on a continuum, and consequently, it may be possible to apply or adapt existing quality of life measures to capture non-health outcomes from genomic testing.

A limitation of our review is that it is restricted to an analysis of information that is in the public domain, typically in the form of PSDs published on the website of the Australian Department of Health. Consequently, our analysis does not capture the perspectives of sponsors, consumers, or the contracted assessment groups regarding the challenges that these stakeholders have faced in demonstrating the value of genomic testing. The systematic collection and analysis of the perspectives of these stakeholders are an important area of future research that could inform the further evolution of MSAC’s approach to the valuation of genomic testing. A strength of our review is that it identifies and systematically organises information from across multiple PSDs (which are not readily identifiable or searchable, despite being publicly available). The fact that two of the authors are members of MSAC is also a strength of the current paper: our familiarity with the ten applications as they have progressed through the assessment pathway means that we are in a unique position to identify emergent themes across the applications.

Whilst some of the findings from the current review are specific to the Australian health system (and all relate to the legislative limits on what can be listed on the MBS), the majority of findings is likely to be generalisable to other jurisdictions. Two findings that are clearly generalisable are (i) the acceptability of relying on diagnostic yield instead of sensitivity and specificity as a measure of test performance and (ii) the acceptability of relying on the cost per actionable variant or carrier couple detected instead of the cost per QALY for assessing cost-effectiveness.

## Conclusions

The need for further evidence of the impact of genomic testing has been comprehensively documented by others: Phillips et al. (2017) identified many evidence gaps in comparative effectiveness research of patient-relevant outcomes for genomic medicine, and Schwarze et al. (2018) have called for more high-quality studies that carefully evaluate the cost, effectiveness, and cost-effectiveness of WES and WGS. MSAC has encountered similar evidence gaps through its consideration of genetic and genomic tests for heritable conditions. Nonetheless, despite these challenges, MSAC has exhibited a willingness to adapt its assessment approaches and accept lower evidence thresholds in particular circumstances. This pragmatic approach has been reflected in a high tolerance for uncertainty, with nine out of ten applications approved for public funding. This tolerance for uncertainty is likely related to the high unmet clinical need and low prevalence of the conditions for which testing has been approved, and the comparatively low rates of likely healthcare resource use associated with the funding approvals. By contrast, MSAC did not accept lower evidence thresholds, and associated higher levels of uncertainty, when an application was associated with a substantial budget impact (NIPT). This trade-off between a willingness to accept uncertainty and clinical need and budget impact is consistent with MSAC’s general approach to HTA applications.

Acceptance of lower evidence thresholds, and higher uncertainty, is likely to have both positive and negative consequences for resource allocation. The key positive consequence of this approach is that particular genomic tests will be funded that would otherwise not be funded, thereby allowing universal access to these tests in areas of high clinical need. A key negative consequence of the approach is that some cost-ineffective tests may be funded. The risk of this occurring can be mitigated if the overall budget impact is considered alongside the estimate of cost-effectiveness, as is MSAC’s usual practice. In other words, standard evidence thresholds can still be required for genomic tests that are likely to be associated with a substantial budget impact.

That said, it is clear that MSAC could further develop its approaches, to account for genomic testing for purposes other than detection of heritable conditions, and for ‘omic’ testing more broadly. As noted by Hoxhaj et al. (2020), it is likely that a single value assessment framework may be too general to apply to all possible clinical scenarios (i.e. screening, diagnosis, prognosis, monitoring, or predictive testing) or to all molecular techniques. This has certainly been the experience of MSAC, where the ‘exemplar/facilitated’ approach for targeted gene panels was not appropriate for carrier screening or identification of congenital anomaly syndromes and was not feasible for large gene panels or WES. Additionally, whilst a focus on clinical management and health outcomes is necessary, it is not always sufficient to fully value the non-health impacts of genomic testing for individuals, their family members, and society more broadly. Research is needed to explore how patient and societal preferences for the health and non-health outcomes of genomic testing (e.g. Mighton et al., 2020; Goranitis et al. 2020) and different evidence thresholds for genomic medicine (e.g. Guzauskas et al., 2019) could be formally incorporated within the value assessment framework used by MSAC for genomic testing.

## Data Availability

The data summarised in this article are available in public summary documents (PSDs) published on the Medical Services Advisory Committee website. A link to each PSD relied on is provided within the article.
